# Opportunities and challenges in clinical learning of midwifery students in public Universities of Tigray Region, Ethiopia, 2020: a qualitative study

**DOI:** 10.1186/s12909-023-04765-5

**Published:** 2023-10-26

**Authors:** Tomas Amare Abraha, Kidisti Tesfay W/tensay, Merhawi Birhane Gebre, Birhanu Abadi Abrha, Gebrhud Berihu Haile

**Affiliations:** 1 College of health science Midwifery department, Mekelle University, Mekelle, Ethiopia; 2 College of health science Midwifery department, Bule Hora University, Bule Hora, Ethiopia; 3 College of health Science School of nursing, Mekelle University, Mekelle, Ethiopia

**Keywords:** Clinical learning, Midwifery students, Opportunity, Challenge, Tigray

## Abstract

**Background:**

Clinical learning focuses on real problems in the context of professional practice in which learners are motivated by its relevance and active participation. Studies showed that midwifery students were challenged by the absence of a variety of cases in non-teaching hospitals, overcrowded teaching hospitals, absence of objective-based evaluation methods, and lack of supervision from clinical instructors. If the theory learned in class was applied in practice, it is helpful to produce skillful and competent midwifery professionals. The aim of this study was exploring opportunities and challenges for midwifery students in the clinical learning environment.

**Methods:**

the study was conducted in public Universities of Tigray, Ethiopia. Phenomenology study design and purposive sampling technique were employed; four focused group discussions and five key informant interviews were conducted. Data were collected using an open-ended guide, transcribed verbatim, entered into ATLAS ti7 software, and translated. Then codes and themes were derived from the transcribed data, and finally analyzed thematically.

**Results:**

a total of 33 participants in which 28 in four focused group discussions and five key informant interviews participated in this study. Based on the result, midwifery students were getting opportunities to practice when they were assigned to non-teaching hospitals, working with close supervision, having smooth relationships with staff, receiving constructive feedback, and evaluated based on their skills. Whereas, they were challenged by aggressive staff, poor follow up, overcrowded teaching hospitals, low usage of skills lab, and short time for clinical practice.

**Conclusion:**

Midwifery students have positive attitude, and were getting opportunities to practice while they were assigned to a very conducive clinical learning environment with supportive and skillful clinical instructors/ preceptors. However, they have negative attitude, and were challenged to work due to the poor attention given to midwifery students’ clinical learning. It is recommended that midwifery students have to practice well in skills lab before they assigned for clinical practice so that the skills lab have to be strengthen with all necessary materials for clinical practice and clinical instructors have to be integrated to teaching hospitals so as to educate students while their hands-on.

## Introduction

According to the international confederation of midwives (ICM), a midwife is a person who has completed a midwifery education program that is based on the ICM essential competencies for basic midwifery practice and the framework of the ICM global standards for midwifery education and is recognized in the country where it is located; who has acquired the requisite qualifications to be registered and/or legally licensed to practice midwifery and use the title ‘midwife’; and who demonstrates competency in the practice of midwifery [1, 2].

The more the quality of clinical learning for midwifery students; the more would be the quality of midwifery care after their graduation. If midwives were learned both the theory in class and the practice in the clinical area, most maternal and infant deaths could be prevented by giving proper maternity and human immunodeficiency virus/acquired immunodeficiency syndrome (HIV/AIDS) services before, during and immediately after pregnancy by well-trained and competent midwives. According to the ICM, the midwifery curriculum should include both theory and practice in the manner of 60% and 40% respectively [1, 3].

Although a care-full examination of the clinical learning environment is helpful, it is not enough by itself. Therefore, strategies and approaches that can improve the clinical learning environment by the health professionals and the community as a whole are needed [4–7].

There is a continuing barrier in improving the quality of midwifery clinical learning which is the mismatch between theory and practice. Students who are competent in the theoretical part are not as competent enough in the clinical practice. As clinical learning is the interactive network of forces influencing learners learning outcomes in the clinical setting, its quality is measured in terms of students learning outcome and competency [8, 9].

The World health organization (WHO) report revealed that a consistent barrier for quality of maternal and newborn care is the issue of poor midwifery educations which lacks practical application. Restrictions to exercise the full scope of midwifery practice and socio-cultural norms; which is against women’s rights, education, and employment. As a result, the preparation of midwifery practitioners and the provision of maternal and neonatal care are variable, across and between low, middle, and high resource countries. The evidence in this report also indicated that midwives, when educated based on the international standards of midwifery, can avert over 80% of preventable maternal and neonatal mortality and stillbirths [2, 10].

The WHO recommended that a midwifery educator should complete a recognized educational program both in theory and practice has legal recognition to practice midwifery, has a minimum of two years clinical experience, and able to research according to his/her level of academic rank. Despite these recommendations, the problem of graduating unqualified midwifery professionals is not solved yet [11].

Poor quality of midwifery training in the clinical area found to be a major impact on midwifery personnel through discrimination by other health providers especially doctors, and cause midwifery professionals to defer clinical decision making to inexperienced junior medical doctors. In addition to this it showed that, even if there is a poor teaching-learning process in the clinical setting, newly graduated midwives are deployed to rural posts without prior clinical experience, supervision or support in dealing with emergency obstetric situations [12, 13].

Midwifery students got it hard to adapt the clinical learning as they felt insecure and frustrated; as a result of a marked difference between the theories, they learned at class and the actual practice the skills performed in the clinical area. In addition to this, students were afraid of performing a vaginal examination and repairing episiotomy during their clinical practice although those procedures were very essential in the delivery room [14, 15].

Although a few studies have been done to find the above gaps in clinical learning of midwifery students in Ethiopia, most of them were done in a single institution and they used quantitative study designs that might not identify the deepest feeling of students in the clinical learning. In addition to this, it was not clear why midwifery students are incompetent? Why they have an unfavorable attitude towards the clinical area? Why they were afraid of performing basic midwifery procedures in the labor ward? Therefore, this study was focused on exploring the opportunities and challenges in the clinical learning of undergraduate midwifery students to fill the above gaps.

## Methods

### Study area and period

This study was conducted at public Universities of Tigray region, Ethiopia. The Tigray regional state is found in the north part of Ethiopia. There are four public Universities in region (i.e. Mekelle University, Raya University, Adigrat University, and Aksum University). Except in Raya University, all public Universities found in Tigray region have health science colleges and midwifery department during the study period. Every year, around 85 to100 midwives were graduating from the three Universities. According to the midwifery departments report,, there were a total of (3rd and 4th year) 63 in Mekelle University, 91 in Adigrat University, and 41 in Aksum University generic undergraduate midwifery students at the data collection time. The study period was from February 1/2020 to February 28 /2020.

### Study design

Phenomenology study design was used to explore the experience and feeling of midwifery students in their clinical learning during the clinical placement period.

### Source and

All year three and year four undergraduate generic midwifery students studying in public Universities of the Tigray region in 2019/2020.

Midwifery professionals and midwifery academic staffs working in teaching and non-teaching hospitals and public Universities of the Tigray region.

### Study population

All selected year three and year four undergraduate generic midwifery students studying in public Universities of the Tigray region in 2019/2020.

Key informants: clinical coordinators, heads of midwifery department, clinical preceptors, heads’ of maternity ward, and senior midwives (worked five or more years in teaching hospitals).

### Sample size determination

The sample size was determined by the level of saturation. Four focused group discussions (FGDs) in undergraduate generic midwifery students and five key informant interviews (KIIs) were used. Each FGD contained 6–8 participants. The time limit for FGDs was 1–1:15 h and for KIIs 30–45 min.

### Sampling technique and procedure

Non- probability purposive sampling technique was employed to recruit study participants. Participants were selected from year three and year four generic undergraduate midwifery students based on their participation (i.e. performance of procedures, skill to perform basic midwifery activities, participation in bed side, rounds and students seminar presentations) during their clinical practice after students’ participation status was obtained from their preceptors and clinical instructors (i.e. clinical preceptors and clinical instructors were used as gatekeepers to get students participation status). Key informants were selected based on their position and work experience. Therefore, both male and female students who actively and passively participate in the clinical practice and key informants based on their position as a clinical coordinator, head department, clinical preceptor, work experience, and place of work were recruited purposively.

### Data collection method

An open-ended English guide was developed and translated to the Amharic language by the principal investigator (PI) with the consultation of the advisors. Two MSc. Students who have experience in qualitative data collection have participated in the data collection process as data collection assistants. One of the data collection assistant was recorded using a tape recorder and the other took keynotes. The PI was moderated the data collection process. FGD took place to explore the students’ shared experiences and KIIs were used for ideas that were not addressed by FGDs and for triangulation.

### Data quality assurance

Open-ended guiding questions were prepared as a leading point and discussed with advisors before the actual work. Training was given to data collection assistants regarding taking keynotes and recording using a tape recorder for one day. An open-ended guiding question was helped to avoid dominant participant. Every day after collecting the data, a debriefing was done by the data collection assistants and the PI. Recorded data were read, re-read, and transcribed by the principal investigator and data collection assistants independently to check the reliability of the data. The collected data were coded by the data collection assistants and the principal investigator independently to minimize personal biases. The authors who didn’t participate in the data collection process did the analysis blindly. Both FGDs and KIIs were taken in a silent place.

### Data analysis and presentation

Data were collected using a tape recorder and key-notes. Then transcribed, entered to ATLAS-ti7, and translated. Codes were created, and categorized then five themes were developed. Finally, thematic analysis method was used, and the result was presented as text and table.

## Result

A total of 33 individuals; by which twenty-eight undergraduate midwifery students in the FGDs (Table 1) and five KIIs (Table 2) participated in the study. The KIIs included clinical coordinator, clinical preceptor, head of the midwifery department, head of labor ward, and senior midwife working in a teaching hospital.

**Table 1:** Socio-democratic characteristics of participants in FGDs

Characteristics		Frequency
University’s name	MU	14
	ADU	8
	AKU	6
Year level	3rd year	7
	4th year	21
Sex	Male	18
	Female	10
Age	20–24	25
	25–29	3
Marital status	Single	28
	Married	0

NB: MU- Mekelle University, ADU- Adigrat University and AKU- Aksum University

**Table 2:** Socio-demographic characteristics of participants in KIIs

Characteristics		Frequency
Institution	AKU	1
	ADU	1
	WGH	1
	ACSH	1
	MU	1
Position	Clinical coordinator	2
	Clinical preceptor	1
	Senior midwifery Professional	1
	Head of midwifery Dep’t	1
Work experience	< 5 years	0
	5–10 years	4
	> 10 years	1
Sex	Male	3
	Female	2

NB: ACSH-ayder comprehensive specialized hospital, WGH- Wukro general hospital

The findings of the result were summarized in to five thematic areas: midwifery students’ attitude towards the clinical learning environment, clinical instructor’s supervision and evaluation methods, health institution staffs role and responsibility, midwifery students’ self-motivation in clinical learning, and the attention given to midwifery students’ clinical learning.

### Midwifery student’s attitude towards the clinical learning environment

This study showed that midwifery students have positive attitude towards non-teaching hospitals and were getting wider opportunities. Because staffs were supportive, there were minimum conflict of interest among students and they have the freedom to work all procedures. They felt like professionals and work freely with the help and close supervision of the midwifery professionals working in the non -teaching hospitals and their clinical instructors.

*“When I was assigned to afflation site for a practical session, I was glad to go and I was interested in working there, no one was influenced me. The staffs encouraged me to work, no medical students were there and I got the opportunity to work freely.”* (FGD, participant C1).

On the other hand, they have negative attitude towards teaching hospitals, and were challenged to work when they were assigned there. Due to teaching hospitals were over crowded with health science students such as residents, interns, health officers, nurses, and the staffs of the hospital. And also, there was high conflict of interest in performing procedures like attending delivery, repairing episiotomy, and performing a pelvic exam. In addition to this, clients become unwilling to be assisted by students due to increased number of students. There was no chance of performing a procedure even observation was difficult for midwifery students because the priority was given to medical students in the teaching hospitals as their clinical teachers were both academic and hospital staffs.

*“I’ve been out for exercise three times so far, there was a big difference between the practices I have done last year at the afflation site in 3rd year with this year’s practice in the teaching hospital. I am forgetting now and there will be a qualification exam at the end of this year that made me afraid of the outcome might be bad. …priority was given to interns and residents. So the leaders should assign midwifery students to afflation site that we would better perform midwifery skill than in teaching hospitals.”* (FGD, participant D1).

However, the health center was favorable clinical area for midwifery students’ clinical learning; there was a shortage of medical equipment. In addition to this, mothers with some complications were referred to higher facilities and midwifery students were working the routine activities. At health centers antenatal care, family planning, and immunization services were high but delivery and postnatal services were almost none.

*“Most of the time, I spent on searching for blood pressure(BP) calf, there is only one BP calf at the ward and if I have a mother who needed frequent measurement, it is difficult to do so….”* (FGD, participant D4).

### Clinical instructor’s supervision and evaluation methods

Although, midwifery departments in the three Universities of the Tigray region have different controlling and supervision systems in clinical learning of midwifery students; this study indicated that midwifery students were working hard when their clinical instructor was working with them and with close supervision. In addition to this, they were highly motivated to develop midwifery skills if the evaluation method was clear and the score was an average of the whole clinical practice session.

*“Midwifery students were happy when their clinical instructor was attending delivery and let them repair episiotomy or to deliver the placenta. They develop self- confidence when the clinical instructor gave constructive feedback on time. I was wondering how every student was motivated to work at such times.“* (A senior midwife from KIIs).

*“I was feeling happy and motivated to work in the clinical practice when my clinical instructor was with me and gave me a constructive feedback. Also, if the evaluation was an average of all days and my clinical instructor put me a grade based on what I was performed instead of one day’s evaluation.”* (FGD, participant F2).

This study revealed that midwifery students were getting opportunities if their clinical instructor was showing and teaching them as his/her hands-on, and were assigned with different clinical instructors; as every instructor can share new skills and knowledge. They prefer if their clinical instructors were changed every week.

*“There was an experienced clinical instructor and he was attending delivery and let me repair the episiotomy, I repaired it well with his support. From that onwards when he came I felt confident and worked hard.”* (FGD, participant C4).

This study found that midwifery students were disappointed when their clinical instructor was absent and took only attendance if she/he came. They felt frustrated and preferred to wait in the corridors rather than working with the staff. It seemed like nobody controls the clinical instructors rather they come if they need to come. In addition to this, there was no clear evaluation method; it was more subjective and they can manipulate it if they need to put good or bad grades. Most students were evaluated not based on the skill they performed but based on the theory they know and the relationship they have had with their clinical instructors.

*“…. Most of the time, we were working without a clinical instructor’s supervision. Clinical instructors came at eleven-thirty to take attendance and on Friday for evaluation. If they got you outside of the rooms at that moment, you will get the least grade; if you were working when they came they trusted you and you will be the higher scorer. Clinical instructors put your evaluation as a snapshot based on one day’s performance. This challenged me to work in the clinical area because whether I was skillful or not I know what my score will be ….”* (FGD, participant A1).

*“…. 2nd -degree holder clinical instructors went for supervision every two weeks and showed, observed, and taught the students and finally evaluate and put their grade. However, the students spent most of their clinical practice time with the health institutions staff; evaluation took place by clinical instructors. Most midwifery students complain about their scores due to its subjective matter. This can be overcome if all universities found in the Tigray region develop similar and clear clinical practice evaluation method….”* (A clinical coordinator from KIIs).

Midwifery students were demotivated by their clinical instructors controlling mechanism. Clinical instructors come and told the hospital staff that they were their students and would be here for clinical practice and went. They didn’t follow their progress daily nor they didn’t know whether the students were improving or not. Students were confused when they perform a procedure without clinical instructors’ supervision and feedback if they were performing the skill-based on the theory they learned or commuting error.

*“…. Most clinical instructors come to the clinical area only one day and left us there and at the end of the time, they put scores without observing us even some of them they didn’t know our face. This made me not to work in clinical areas rather I prefer to go to the dorm or to play games at the empty rooms….“* (FGD, participant E1).

### Health institution staffs role and responsibility

This study found that midwifery students would love to go to a health facility with supportive and role mode staff. They spend most of the time with the hospital staff if the health institution staff showed and let them practice and gave constructive comments. According to this study, some midwifery professionals working in health centers and general hospitals were happy when students went there for clinical practice because they were small in number and did every activity by themselves and were much tiered. Due to this students got the chance of developing midwifery skills and the staff gave support and feedback on time.

*“When the health institution staffs showed me how to perform the procedure and let me practice on it while they were assisted me and gave me feedback I preferred to work all days with such staffs….“* (FGD, participant A2).

*“…… we as midwifery professionals tried to show students when they come for clinical practice to develop midwifery skills and orient all staffs to allow students to observe, assist and perform such skills and to work in a coordinated manner with residents, interns and other health science students, though all staffs might not apply it….“* (Head of labor ward from KIIs).

Midwifery students needed to practice at health centers especially in family planning, antenatal care, and immunization service units. The staff enforced students to develop their clinical skills in injection, insertion or removal of implants, and to take a full history and to perform a complete physical examination in real clients with closed supervision and control.

*“… When I was assigned to the health center I wake up early in the morning like an employee and went there on time…. Sometimes I was there before the staffs arrived. The clients looked at me like professional midwifery and the staff let me perform all procedures. Now I am skillful in family planning and antenatal care services….“* (FGD, participant A4).

On the other hand, students were challenged to go to health institutions with aggressive, non-supportive, and careless staff. Some staff was treating them like they know everything and angry at them if they made errors. Students wanted to observe all activities which were performed at the health institutions but they complained that some staffs were ordered them to be outside of the room while they were performing complicated procedures like repairing the perineal tear, managing post-partum hemorrhage and resuscitating neonates.

*“As a mother developed postpartum hemorrhage in the delivery room a staff ordered me to go out and closed the door inside. Even if, most complicated cases were referred to higher health facility, the staffs’ didn’t let me even to observe how to manage and how to refer…”* (FGD, participant B4).

This study showed that some health institution staffs were doing procedures wrongly and were unable to explain why they were doing so. Some procedures were performed totally different from the theory the students have learned in class and there was no chance of asking questions and made students confused.

*“…in class, we have learned the theory but it was not clear and when we went to clinical practice it was totally different. The staffs didn’t follow the steps that we have learned and if we ask questions, they were not willing to answer rather they put us under blacklist and we felt guilty that our clinical instructor might punish for that….“* (FGD, participant D4).

### Midwifery students’ self-motivation in clinical learning

This study found out that midwifery students were motivated when assigned to the clinical learning environment, as clinical learning is much memorable than the theory they have learned in class. They have the motive of helping mothers especially those who come from rural areas. They were eager to know new procedures they didn’t observe or performed before to develop their skills to manage complications at places where they might be assigned after graduation. They also need to be more competent than their classmates and that the opportunity of working with the supervision of the clinical instructor will never come again after they have been graduated.

*“….the clinical learning that I am learning since the last two weeks is more memorable and useful than the theory that I have been learned in the past six months…. There is nothing better than helping mothers that made me happy and motivated….”* (FGD, participant B4).

Midwifery students were motivated to work in the clinical learning environment due to the midwifery profession is linked with two lives. They were interested in saving the mother’s and her baby’s lives which made them happy and satisfied. In addition to this; students were interested in their clinical learning to put their contribution in decreasing the current maternal and neonatal death happening in Ethiopia as they have graduated.

*“…. What motivated me to work hard in the clinical practice was the maternal and neonatal death I have heard and I am watching in my country, Ethiopia and to put my fingerprint in decreasing this tragedy….“* (FGD, participant A3).

This study revealed that students were eager to work in clinical practice when they were faced with complicated cases in health institutions. They need to know new procedures that they didn’t saw it before and when the health workers were doing procedures wrongly to the clients in order not to be unskilled. In addition to these, they were motivated to work at their utmost capacity to minimize future problems they might face and to help mothers in need (pain).

*“I try to know as much as I can to help mothers in need, on the other hand, when unskilled health professionals did procedures wrongly on clients; I need to work hard not to be like him//her.* (FGD, participant E1)

However, midwifery students believed that being working in the delivery room contradicted with their religion. If they were attended delivery they didn’t want to go to church. They believe that a person who attended delivery is not allowed to enter into the church which they have heard from the community and religious leaders. In addition to this, students were out of work if one of their classmates were absent. Some times when clients were unwilling to be examined by students, they preferred to go home instead of convincing the clients.

*“…. If you are an orthodox religious follower, it is not allowed you to enter to church if you were attended delivery which made us absent from work or church….“* (FGD, participant C2).

### The attention given to midwifery students’ clinical learning

This study indicated that even if having a clinical preceptor was crucial for clinical learning of midwifery students and help to produce skillful and competent midwifery professionals; it was misused. Midwifery students complained that there was a big difference in qualification among clinical preceptors assigned at different afflation sites and some of them were not able to answer questions raised by students. The students stressed that clinical preceptors should be changed every week which will help to get new skills and knowledge for students.

*“…although, clinical preceptor was important for midwifery students; there was a qualification difference among clinical preceptors: some of them were master’s holders while the others were degree and diploma holders. A clinical preceptor should be at least one degree higher than his students. Sometimes questions raised by students were not answered by clinical preceptors due to shortage of knowledge on the subject….”* (FGD, participant A1).

On the other hand, there was no clear evidence if using clinical preceptor was better than using clinical instructors for clinical learning of midwifery students. If the clinical preceptor who was assigned and agreed with the department was absent nobody cared for the students which indicated a conflict of interest among the staff members. And also, the departments found in different Universities of Tigray were not shared experiences related to the use of clinical preceptors.

*“….we didn’t have clinical preceptor yet because there was no evidence that showed students who have learned with clinical preceptors are more skillful and competent. And also we believe as a department all staff members haven’t the same qualification and if assigned someone there might be a conflict of interest among the staff members which lead to poor clinical learning of our students. We are teaching them by our clinical instructors who have one and more years of clinical experience. This can be checked by experience sharing among the departments with the clinical preceptor and those without it….“* (Head department of midwifery from KIIs).

This study found out that there was a poor controlling system of the department to the clinical instructors. Some clinical instructors were assigned to clinical practice while they have theoretical class and most of the times were absent or come once a week for evaluation purposes. As the clinical instructors absent, nobody controls the students, and they were out of work or playing games with friends. Even if there was a clinical coordinator assigned to supervise the clinical teachers and students; there was no action taken yet to the clinical instructors which indicated that poor attention is given to midwifery students’ clinical learning.

*“…. When the department assigns clinical instructors it should be sure that the instructor has finished the theoretical class otherwise it may not have value if the instructor is assigned to theory and clinical practice at the same time. That’s why some clinical teachers were absent from clinical practice….”* (FGD, participant B1 said by increasing her voice).

Even if assigning midwifery students to the afflation sites is crucial, participants disagreed on the time allocated to clinical practice for midwifery students which was too short. Students were assigned to different units in hospitals and allowed to work for three or fewer weeks. The time allocated was finished and ordered to rotate before students were performed the minimum procedures required and made students confused.

*“…. The time allocated for clinical practice was not enough let alone for practice it was too short to observe. Especially at the afflation site, we were assigned to six or more units and the time was over before we perform even one procedure…”* (FGD, participant D1).

*“….The time allotted for a practice session was not enough because as the students were observing and assisting us, the time allowed for that unit will over and they will switch to another unit just before they perform a procedure by themselves….”* (A clinical preceptor from KIIs).

This study revealed that there was no clear objective whenever midwifery students were assigned to clinical practice. They didn’t know the objective of the attachment, their scope of practice at a specific unit was unknown. No clinical instructor gave them any guiding material or course outline. There was little or no orientation before they went to the clinical learning environment. The students didn’t know about the evaluation method and who will evaluate them and what they should and shouldn’t do in the clinical practice environment.

*“We have no idea when we went to the afflation site. We didn’t know what we were going to do. There was no guidance given to us, and we were confused about what type of activity with whom to perform.”* (FGD, participant C4).

This study showed that midwifery students were not practiced well in the skills lab before they went to the actual clinical practice. This challenged them at times they need to examine clients in the clinical areas. Students were afraid of performing pelvic exams in the labor ward because they didn’t practice it much in the skills lab using dolls which indicates the attention given to midwifery students’ clinical learning is poor.

*“….Even when I went to the hospital, I didn’t work well in the skills lab until I went straight to a patient and I fear that I will make errors.”(FGD*, participant A4*)*.

Participants explained that the amount of birr given during their assignment to the afflation site was not matched to the current market inflation and were suffered from the economic impact. This challenged midwifery students during their clinical learning sessions.

*“The amount of birr paid for all students is not enough compared to the current market value of goods. While I was in the ward, I was thinking about what type of food I should eat at lunch and dinner because the birr that is paid by the university was not enough…… I was counting the number of days left to return to campus.”* (FGD, participant B3).

Participants complained that most midwifery students and clinical instructors were not vaccinated to hepatitis and this challenged them to practice activities for fear of contamination. Even if they have been complaining for the past three years they were not vaccinated yet.

*“… As we were working in health institutions with the staffs we should be vaccinated for hepatitis because we are vulnerable to contamination due to our limited clinical experience…. There were many patients with unknown status I was examined because the laboratory result comes later. I was worried about….“* (FGD, participant F2).

In addition to this, the arrangement of courses is disorganized. Theoretical courses that should be given in the second year were given in the third year which made midwifery students confused.

*“Courses should be arranged in a logical order: For example, it is hard to learn about antenatal care before learning nutrition course, how can I advise pregnant mothers about nutrition education? How can I give infection prevention education for clients before I have learned the environmental health course? These should be re-organized for future midwifery students….”* (FGD, participant C4).

## Discussion

This study explored the opportunities and challenges in clinical learning of undergraduate midwifery students related to midwifery student’s attitude towards the clinical learning environment, clinical instructors’ supervision and evaluation methods, health institution staffs role and responsibility, students’ self-motivation, and attention given to midwifery students’ clinical learning.

This study showed that Midwifery students have positive attitude, and were getting wider opportunities to practice while they were assigned to non-teaching hospitals. Because staffs were supportive, there was a minimum conflict of interest among students and they have the freedom to work. This study contradicted with a study done in Iran. Most midwifery students have negative attitude and faced limited opportunities when they were placed in non-teaching hospitals. Because, non -teaching hospitals are not student oriented, staffs are unwilling to teach, and as a result midwifery students made to do ward chores rather than practicing midwifery skills [16]. This could be due to differences in staff willingness and attitude towards teaching students.

Midwifery students have negative attitude, and were challenged to work when assigned to teaching hospitals. Teaching hospitals were overcrowded with health science students such as residents, interns, health officers, nurses, and midwifery as well as the staff of the hospital. There was high conflict of interest in performing procedures like attending delivery, repairing episiotomy performing a pelvic exam, and clients become unwilling to be assisted by students due to the increased number of students. There was no chance to perform a procedure even observation was difficult for midwifery students because the priority is given to medical students. This study is similar to a study conducted globally by ICM; when midwifery students were assigned to a clinical area with medical students, more opportunity is given to medical students. Midwifery students are forced to work at nights and weekends with little or no clinical supervision [17] and Iran, midwifery students face a conflict with obstetrics residents who also need to experience attending normal vaginal births which decreases the confidence of students [16].

However, the health center was favorable clinical area for midwifery students’ clinical learning; there was a shortage of medical equipment. In addition to this, mothers with some complications were referred to higher facilities and midwifery students were working the routine activities. At health centers family planning and immunization services were high but delivery and postnatal services are almost none. This result is supported by a study conducted in Ethiopia and Iran; absences of a variety of cases in clinical sites other than the teaching institutions make students perform the routine procedures rather than gaining new and complicated skills. In some clinical sites especially in health centers, midwifery students may finish the time allocated to clinical practice without attending the minimum number of normal delivery [18, 19].

Midwifery students were working hard and highly motivated to develop midwifery skills if the objective and evaluation method was clear and be evaluated based on common criteria and the skills performed instead of the theory they know. This result is in line with a study conducted in Ethiopia; if the objective of being in the clinical site is known by the midwifery students and all instructors have evaluated based on common criteria for all students; they (midwifery students) become motivated to learn in the clinical practice during their stay [18].

This result revealed that midwifery students were getting opportunities if their clinical instructor was skillful in performing procedures, was showing and teaching them as his/her hands on. This result is in line with a study conducted in Iran, which indicated that the most fundamental skill for the clinical educator is the application of scientific-based practice. Clinical instructors are expected to apply in practice what they have said in class and explain why they are performing it which motivate students [20, 21].

This study indicated that midwifery students were confused when they perform a procedure without clinical instructors’ supervision and feedback if they were performing the skill-based on the theory they learned or commuting error. This result is supported by a study done in and Iran Malawi, midwifery students were worried that they could not achieve the minimum midwifery experiences before the end of the attachment while they were doing procedures without supervision due to fear of commuting errors [16, 20, 22].

This study found that midwifery students would love to go to a health facility with supportive and role mode staff. They spend most of the time with the hospital staff if the health institution staff showed and let them practice and gave them constructive comments. This study is similar to a study conducted in Malawi and Iran; the Head department and staffs are expected to support and to be a role model for midwifery students by showing procedures step by step instead of assigning a few responsible staff. The secure and favorable clinical environment made students focus on their clinical education and it is a must [21, 22].

This result indicated that midwifery students complained, some staffs’ were ordered them to be outside of the room while they were performing complicated procedures like repairing perineal- tear and resuscitating neonates. This is in line with a study conducted in Belgium and Iran; in the case of pathologies like post-partum bleeding, preceptors and staffs didn’t explain what is going to be done and why rather they pushed aside for the students that lead midwifery students not to participate in such acute and real situations and make them demotivated to learn [19, 23].

Midwifery students mentioned that some health institution staffs were doing procedures wrongly and were not able to explain why they were doing so. Some procedures were performed totally different from the theory the students have learned in class and there was no chance of asking questions and made students confused. This is supported by a study done in Iran and Malawi; Midwifery students faced challenges of the theory-practice gap during their clinical practice. There was a difference between the theory learned in class and the actual practice done in the patients. Even every midwifery professional did the skills differently and if students ask why, the staff and their instructors’ reply to do as they were doing. This made midwifery students to be confused and lose their confidence [16, 20, 24].

Midwifery students complained that there was a big difference in qualification among clinical preceptors assigned at different afflation sites and some of them were not able to answer questions raised by students. The students stressed that clinical preceptors should be changed every week which will help to get a new skill and knowledge for students which is supported by a study done in Spain; there were differences among clinical preceptors qualification and students need to be assigned to different clinical preceptors to gain enough clinical skills [25]. But contradicted with a study conducted in Belgium and Ireland; students stressed the need for continuity of clinical preceptor during clinical placements. This helped midwifery students to have trust, create connections, and to develop professional relationships which in return help them to see their progress based on their clinical preceptor’s follow up and constructive feedback [23, 26]. This might be due to differences in assignment, qualification, and using of clinical preceptor.

Participants disagreed on the time allocated to clinical practice for midwifery students which was too short. Students were assigned to different units in hospitals and allowed to work for three or fewer weeks. The time allocated was finished and ordered to rotate before students were performed the minimum procedures required and made students confused. This is supported by a study done in Spain, Ireland, and Ethiopia; most midwifery students stressed that the curriculum should give more time to clinical practice than theory. Students who have good achievements are faced with challenges in the clinical practice due to that they spent more time on classroom learning. In some clinical areas, even the schedule can be passed without performing simple procedures and usually without watching any case [18, 25, 27].

### Limitations

Since this study was done at public Universities only, it might not applicable to midwifery students learning in private Universities of Tigray region.

## Conclusion

Midwifery students have positive attitude, and were getting opportunities to practice while they were assigned to a very conducive clinical learning environment with supportive and skillful clinical instructors/ preceptors.

However, they have negative attitude, and were challenged to work due to the poor attention given to midwifery students’ clinical learning. It is recommended that midwifery students have to practice well in skills lab before they assigned for clinical practice so that the skills lab have to be strengthen with all necessary materials for practice, the time allocated for practice have to be increased to four weeks per unit of practice and clinical instructors who are only academicians have to be integrated to teaching hospitals so as to educate students while their hands-on.

## Data Availability

The datasets generated and/or analyzed during the current study are not publicly available due to the involved private information of the individual participants, but they are available from the corresponding author on reasonable request.

## List of abbreviations

FGD: Focused Group Discussion
HIV/AIDS: Human Immunodeficiency Virus/ Acquired Immunodeficiency Syndrome
ICM: International Confederations of Midwives
KIIs: Key Informant Interviews
PI: Principal Investigator
WHO: World Health Organization
